# The use of CT scan imaging technique to determine pear bruise level due to external loads

**DOI:** 10.1002/fsn3.882

**Published:** 2018-11-20

**Authors:** Mohsen Azadbakht, Mohammad Vahedi Torshizi, Mohammad Javad Mahmoodi

**Affiliations:** ^1^ Department of Bio‐System Mechanical Engineering Gorgan University of Agricultural Sciences and Natural Resources Gorgan Iran

**Keywords:** bruise, CT scan, non‐destructive test, pear, storing

## Abstract

X‐ray computed tomography (CT) is an effective noninvasive tool to visualize fresh agricultural commodities’ internal components and quality attributes, also imaging via X‐ray CT is a non‐destructive and well‐developed method applied in measuring the internal effects of agricultural products. In the present research, 120 healthy pears with their health verification through the CT were selected. Next, 81 healthy pears were selected and subjected to quasi‐static and dynamic loading. The impact of the incoming pressures was investigated within 5, 10, and 15 days of storage. After loading and storing with the use of CT method, the total volume and the bruise volume of the pears were measured and the ratio of the bruise volume to the volume of each pear was calculated. Quasi‐static loads were pressurized over a period of two ways; the pressure of wide edge was exerted at three force levels of 70, 100, and 130 N while the pressure of the thin edge was applied at 15, 20, and 25 N. Dynamic loading was performed by utilizing a pendulum and 300, 350, and 400 g mass. The results of the experiments indicated that in the quasi‐static loading, the maximum and minimum amounts of pear bruise were 45.138% and 0.094% of the fruit, respectively. Besides, in the case of thin edge pressures, the minimum and maximum bruise levels were 0.007% and 19.88%, respectively. These values were obtained through 5 and 15 days of storage, respectively. In the dynamic loading, the maximum and minimum amounts of pear bruise were 47.36% and 0.21% of the total fruit, respectively, occurring at 400 and 300 g mass impact.

## 1 INTRODUCTION

A crucial aspect of distinguishing fruits from other products is their rapid destruction. The high content of water and carbohydrates entails metabolic processes along with the growth of microorganisms, leading to qualitative and quantitative damages. These products are particularly vulnerable to be damaged from scratches and impacts. Accordingly, it is necessary to apply well‐developed non‐destructive methods that provide insight into qualitative features in the early stages of the post‐harvest process in the agricultural sector (Hernández‐sánchez, Moreda, & Herre‐ro‐langreo, 2016). The non‐destructive quality assessment of agricultural products has become a significant topic for the agricultural industry (Kotwaliwale et al., 2014). Over the past decade, non‐destructive methods have been utilized and preferred over destructive techniques in evaluating fruit quality. Such examinations allow for the individual measurement and analysis of fruits and reduce the losses as the product is not mechanically injured. Furthermore, by repeating the same measurements over time, more suitable results can be obtained from an agricultural product. An increase in consumer demand to ensure the quality of internal and external products and the interest in new industries resulted in the development of fast and cost‐effective non‐destructive tools for detecting and monitoring the fruit quality (Arendse, Fawole, Magwaza, & Opara, 2018). The use of CT and X‐rays as non‐destructive methods may allow checking the bruises on fruit (Diels et al., 2017). Pear (*Pyrus communis* L.), a valuable fruit, is consumed in various forms such as jam and is dried because of its special aroma and sweetness (Kolniak‐Ostek, 2016; Pérez‐Jiménez & Saura‐Calixto, 2015). It contains considerable amounts of sugar, vitamins, organic acids, polyphenols, minerals, and other nutrients. Free sugars, organic acids, free amino acids and fatty acids, minerals, and perfumes are natural components of many fruits and vegetables and they play important roles in preserving the quality of the fruit and determining its value (Chen, Wang, Wu, Wang, & Hu, 2007; Yi et al., 2016). Moreover, consuming healthy fruits reduces the risk of chronic illness as they contain numerous antioxidants (Qu, Zhao, Zhao, Liu, & Yang, 2016). Mechanical damage to fruits is mainly caused during the harvest operations; however, in packaging and sorting stages, during transportation, and in the consumer market, retailers and consumers pose mechanical damage to the fruits that can reduce their quality (Li & Thomas, 2014; Opara & Fadiji, 2018). Mechanical damages result in economic losses. In order to minimize these losses, certain studies are conducted to investigate the impact loading and quasi‐static loading conditions during and after harvesting fruits, vegetables, and other biological materials (Stropek & Gołacki, 2015). Some researchers asserted that a slight impact force may not entail any changes in the fruit. On the other hand, when you see a portion of the fruit damage during a physical injury, there is obviously a significant bruise during the warehousing process. Physical evidence also includes cell fractures and color changes in the desired fruit that occur when each cell is squeezed. Such damages eventually ruin the fruit and change its color (Opara & Pathare, 2014).

Several attempts are made to find the suitable methods for assessing the qualitative characteristics of agricultural and food products without any destructive consequences. Advanced technology has broadened the horizon to determine the quality of non‐destructive nutrients through some techniques such as X‐ray imaging, computed tomography (CT), magnetic resonance imaging (MRI), and ultrasound techniques (Kotwaliwale et al., 2014).

Razavi, Asghari, Azadbakh, and Shamsabadi (2018) applied MRI technique by following a static loading in order to assess the pear bruise and found that time and force, both individually and simultaneously, have a significant effect on the bruise volume. They also reported that the best time to consume pear after loading, discharge, and internal bruises during harvesting and storage is 12 days.

Kim, Kim, Park, Kim, and Cho (2014) indicated that infrared lock‐in thermography and the information it provides are of great assistance to detect the mechanical damage to the fruit, particularly in the early stages of bruising.

Zhang, Cui, and Ying (2014) employed a vibratory acoustic non‐destructive measurement in order to investigate pear tissue. They selected three tissue indexes (MF, FF, and Stiff) and seven vibration parameters (F2, A2, P400, P800, P1200, P1600, and EI) from the samples. They indicated that, compared with MF and FF, Stif is a more proper indicator of the evaluation of the qualities of the fruit and is well correlated with the vibrational parameters. They also expressed that the multiple vibration parameters provided more information for predicting the tissue by the unit vibration parameter. Following correction via SI, the performance of the prediction model was improved. The results of the experiments revealed the significance of both the original and modified prediction models. Furthermore, the evaluation of pear tissue was combined with the LDV method which rendered the proposed modeling feasibly.

Muziri et al. (2016) studied microscopic analysis and tissue detection in pears by employing X‐ray computed tomography and confirmed that the tissue of crispy fruits is more porous than non‐crispy ones. They further provided evidence regarding the formation of lysigenous masses in crispy pears and indicated that these fruits are larger and more oval while non‐crispy fruit cells are more spherical.

In their study on bruises in apples, Diels et al. (2017) applied a computerized X‐ray tomography and reported the bruises created by a spherical impactor (pendulum) using the buccal visualization in all 2D and 3D images. This bruise can be highly irregular, suggesting that the estimates of bruises based on simple geometric hypotheses cannot provide accurate results.

During transportation, the pear is placed under the two pressures of wide edge (from pears, vehicles, Pear box etc.) and thin edge pressure (pedicle a pear on another pear, the sharp side of the pear box, etc.), as well as the possibility of being under the impact load (when picking from the tree, sorting machines, etc.). In the current paper, these loading forces are simulated to determine the rate of pear bruises after loading and storage. The present research was conducted to evaluate pear bruises by quasi‐static and dynamic loading over different periods using CT in a way to calculate the storage time of pear fruit. It was indicated that through appropriate measures, post‐harvest problems such as bruises and economic losses can be reduced prior to, during, and following the harvest.

## 2 MATERIAL AND METHODS

### 2.1 Sample preparation

The Pears (Spadana variety( were purchased from gardens around Gorgan, Golestan province, Iran. Samples were placed in separate boxes, and inside the boxes, pears were at a distance from each other. The life of the pear tree used in this research was 5 years old. The days after full bloom was about 120 day, and the pears were freshly harvested and the experiments were carried out as quickly as possible. Then Samples were taken to the laboratory of Gorgan University of Agricultural Sciences and Natural Resources. They were placed in an oven and their moisture contents were measured (Azadbakht, Torshizi, Ghajarjazi, & Ziaratban, 2016). The calculated moisture content of the pears was 77.92%. Environmental conditions for testing were conducted at a temperature of 18°C and relative humidity of 72%.

### 2.2 Quasi‐Static test

**Figure 1:**
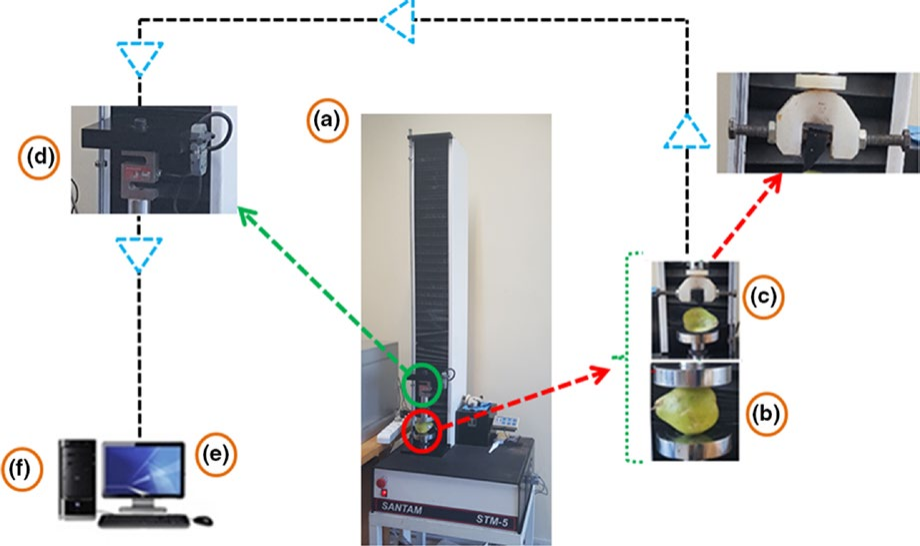
Static quasi‐load diagram of pear. a: The force‐deformation device (Indestrone); b: Jaw wide edges; c: Jaw thin edges; d: Load Cell; e: Computer; f: Information Extract

In order to perform the wide and thin edge compression mechanical test, a pressure‐deformation device (the Santam Indestrone ‐STM5‐Made in Iran) with a load cell of 500 N was employed. The compression test, where the two circular plates were utilized, was performed at a speed of 5 mm/s with three forces of 70, 100, and 130 N and three repetitions. In this experiment, the pear was horizontally placed between the two plates and pressed with the duration of the recorded measurement. Regarding the thin edge compression test, a double‐jaw of plastic with a rectangular cross‐section dimension of 0.3 × 1.5 cm was designed. The test was performed at a speed of 5 mm/s with three forces of 15, 20, and 25 N and three repetitions (Figure 1).

By moving the movable jaw, the pressure operation was carried out until the force reached the desired level (Azadbakht, Vehedi Torshizi, Ghajar Jazi, & Ziaratban, 2016).

### 2.3 Impact test

**Figure 2:**
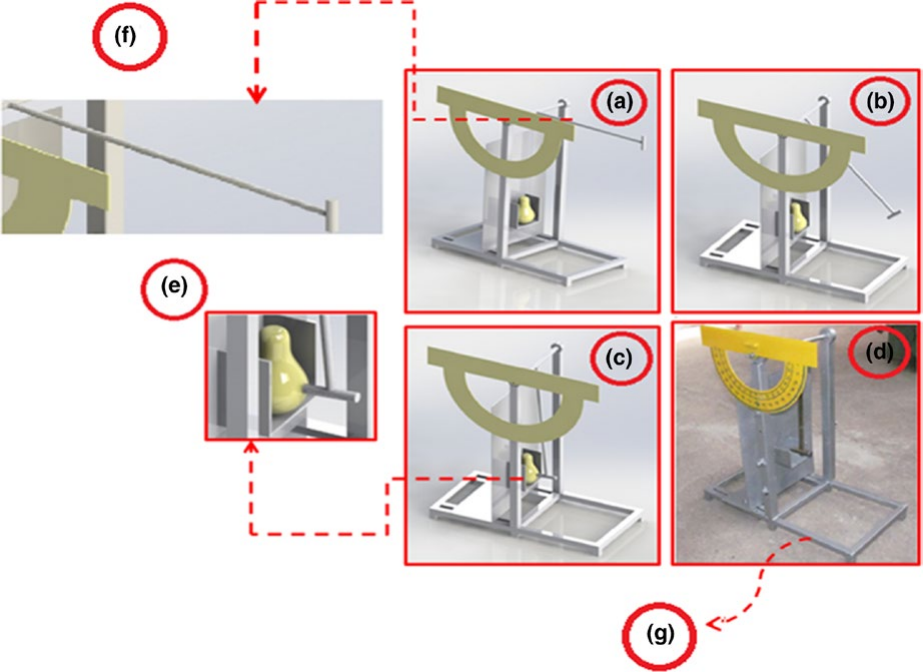
Schematic of the impact machine. a: Pendulum at a 90° angle; b: Walking along the path; c: Collapse pendulum to pear; d: Main device profile; e: Place the pear; f: Pendulum blow; g: the base of the device

First, the pendulum and the required masses were made in a workshop in Gorgan Biosystem Mechanics Group (Figure 2). The fruits were placed in the desired position and then the device arm (arm length = 33.5 cm) was raised to the desired angle (90°), and in the controlled state of the arm impacted the pear. The pendulum had a 200‐g arm and three different attachment masses of 100, 150, and 200 g for knocking. It should be noted that air resistance and friction were neglected through this procedure.

### 2.4 Pear preparation for imaging

In the present experiment, 120 pears were selected based on a non‐destructive CT scan. Next, 81 pears without any bruises were selected. Following the dynamic and quasi‐static loading, the pears were stored for 5, 10, and 15 days. The storage conditions were similar to those of sale centers so that the fruits could be studied during storage and consumption. The ambient temperature was 14°C and the relative humidity was 66%.

### 2.5 Imaging via CT scan method

**Figure 3:**
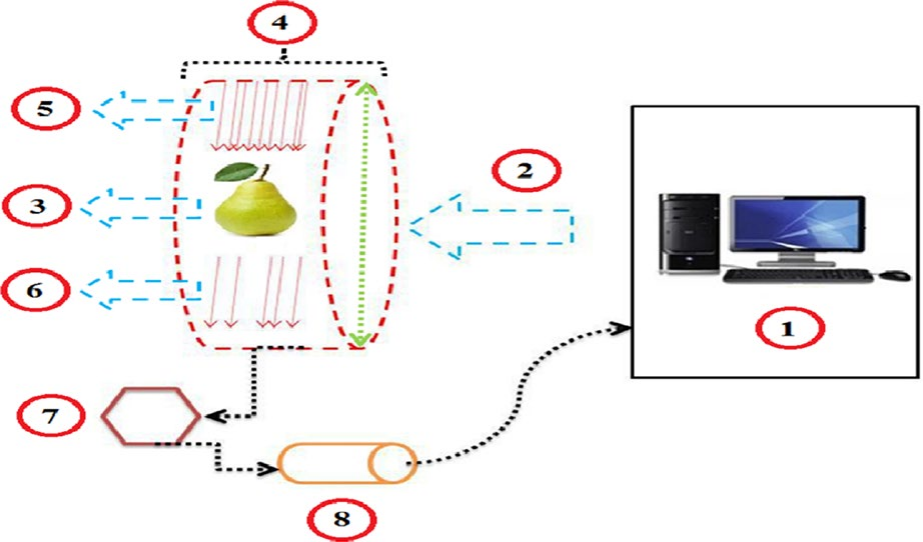
CT scan imaging process. 1‐Control Room; 2‐Send information and Setting Device; 3‐Pear sample; 4‐X‐rays tube; 5‐X‐ray input; 6‐X‐ray outlet from sample; 7‐Crystals CT Scan; 8‐Converted to image codes

To perform the imaging, pears were taken to the test site and inserted into the CT scan crate after starting CT scan through the control room (Figure 3, No. 1; Figure 3, No. 3) and through X‐rays tube (Figure 3, No. 4) X‐rays were drawn on pears (Figure 3, No. 5). Some of the energy was absorbed by the pears, and the rest of the rays were rejected (Figure 3, No. 5 and No. 6); the light shades of the pear were absorbed by the crystals contained within the CT scan (Figure 3, No. 7). Afterward, using a light converter, they were converted to image codes (Figure 3, No. 8) and delivered to a computer room to reproduce images. Figure 3 illustrates the process of CT scan imaging.

**Figure 4:**
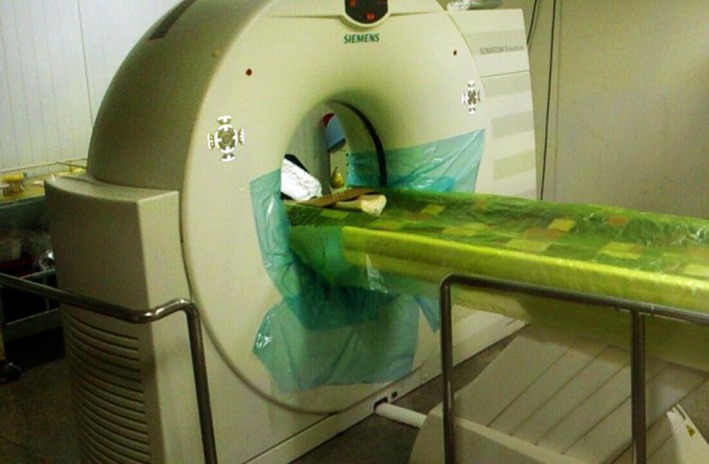
The CT Scan device used in the research

Then, 5, 10, and 15 days after the quasi‐static and dynamic loading, each pear was scanned with the Siemens Computed Tomography (CT) Scans of the SOMATOM Emotion 16‐slice model, made in Germany. This device is a third‐generation CT device in which the tube and detector are placed opposite to each other 360° round the pears in a series of turns to create the image. Also, the pitch was locked for the test; that is, pitch 1. Images were recorded at 80 kV and 120 mAh current, and 1 mm slices were used to create full images. The images created by the Syngo CT 2012 software were recorded and extracted in the form of two‐dimensional and grayscale images. Convolution kernel, which demonstrates image resolution level, was B31Smooth and the images were created by 512 × 512 matrixes. Figure 4 illustrates the CT scan used in this experiment.

**Figure 5:**
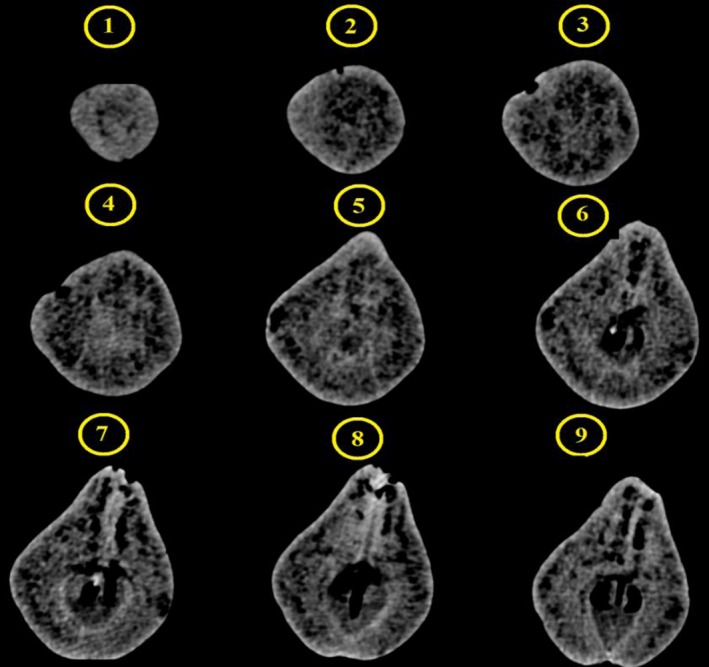
CT scan imaging process. There are 1–9 steps in creating the image, 1 first image and 9 final images

**Figure 6:**
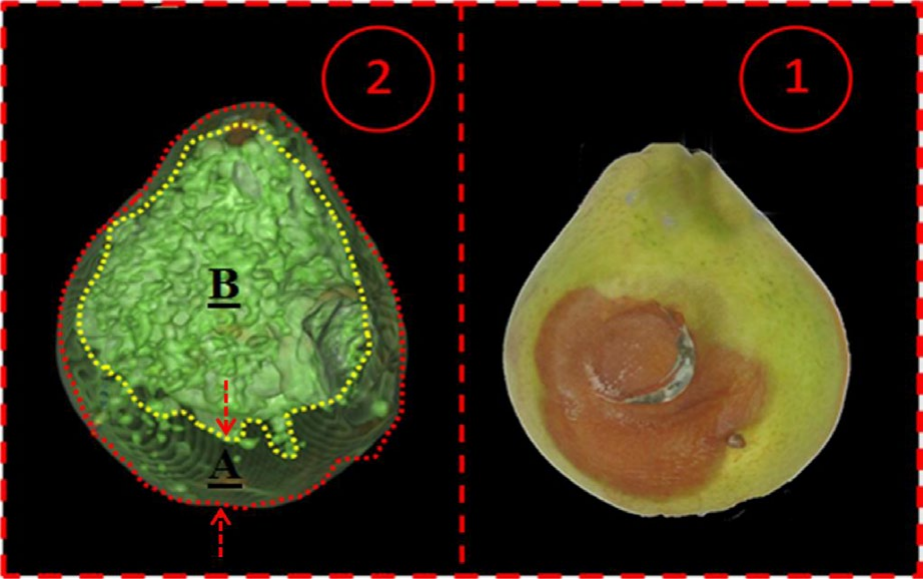
Two‐dimensional view of pear prior to and following image processing. 1. Bruise in the real image; 2. Bruise in the processed image (A: Bruise part in pear; B: healthy part of pear)

An interval was applied between bruises and imaging in order to allow the bruises to reduce their moisture content and be better fixed on the fruit. Such a difference in moisture can increase the absorption of X‐rays between healthy and unhealthy texture (Diels et al., 2017). Subsequently, using the data obtained by the device, the total volume, bruise volume of each fruit, and the two‐dimensional color images of each bruise were measured; the ratio of bruises to the total volume of each pear was obtained by CT scan and was recorded with loading time in the Excel software. Furthermore, during the imaging of each pear, 70–100 images on average were captured to achieve the full‐size pears for 3D reconstruction. The steps involved in this process for each image are indicated in Figure 5. A redesigned two‐dimensional image of the pear is also depicted in Figure 6, which can be divided into healthy texture and rotten texture. In Figure 5, No. 1 and No. 2 present the bruise location and the image created by the CT scan, respectively.

### 2.6 Statistical analysis

Samples were stored for 5, 10, and 15 days after quasi‐static and dynamic loading, followed by the imaging process. All the experiments were performed in three replications and the results were analyzed by employing a factorial experiment in a completely randomized design with SAS statistical software.

## 3 RESULTS AND DISCUSSION

**Figure 7:**
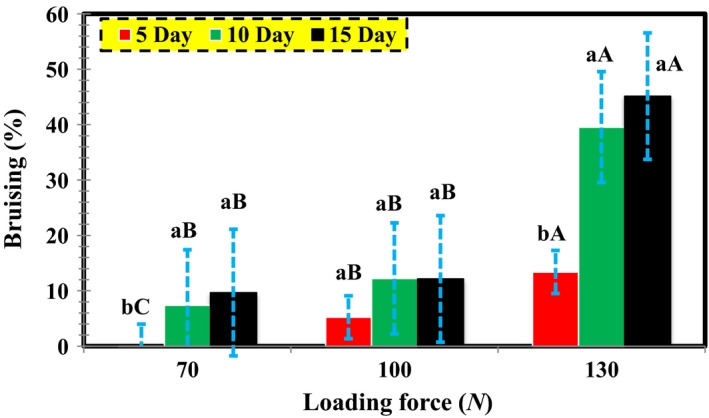
The mutual effect of loading force and storage period on bruise percentage regarding wide edge pressure

**Figure 8:**
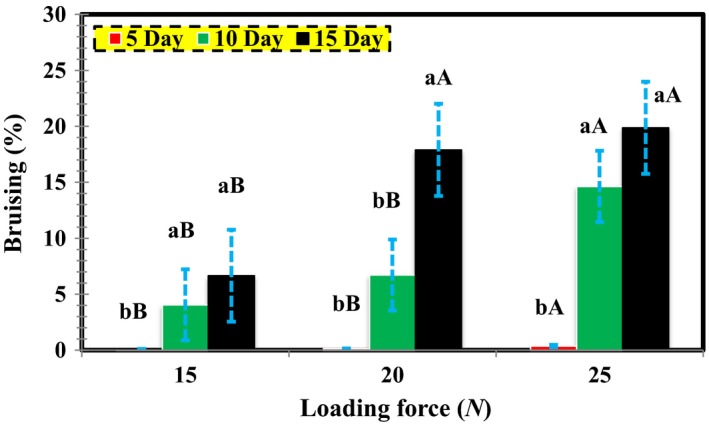
Mutual effect of loading force in storage period on bruise percentage concerning thin edge Pressure

**Figure 9:**
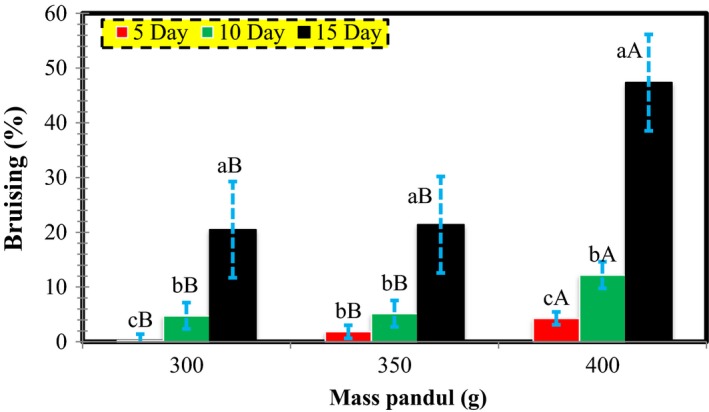
The interaction of loading pressure with storage period and its influence on bruise percentage in the impact mode

Table 1 presents the analysis of variance (ANOVA) results for the effect of loading force and storage period on the percentage of bruises (volume of bruises per total pear volume) as far as the wide edge, thin edge, and impact pressures are concerned. As it can be observed, there exists a statistically significant relationship between storage time and loading force in all three loading modes at 1% level. Additionally, the effect of the storage period on the loading force was significant in all loading modes at 5% level. Accordingly, the average operation was performed with the LSD test, with the results illustrated in Figures 7, 8, 9.

**Table 1:** The analysis of variance of pear bruises under the loading and storage period

Static loading					
Parameters	*df*	Mean square		*F* value	
		Wide edge pressure	Thin edge pressure	Wide edge pressure	Thin edge pressure
Storage period	2	670.900	482.74	14.370**	34.81**
Loading force	2	1,898.86	146.48	40.67**	10.56**
Storage period × Loading force	4	156.95	447.93	3.36*	3.46*
Error	18	46.68	13.86		

Dynamic loading			
Parameters	*df*	Mean square	*F* value
Storage period	2	1,936.44	11.78**
Loading force	2	456.009	8.65**
Storage period × Loading force	4	152.97	1.65*
Error	18	48.75	

*,** denotes the significant difference at 1% level (p < 0.01) and at 5% level (p < 0.05) respectively.

### 3.1 The effect of wide edge pressure on bruise percentage

As illustrated in Table 1 and Figure 7, there is a significant relationship between the pressure of wide edge in storage periods and the loading force at 1% level. Based on the findings, at a constant loading force, there was a positive relationship between the storage period and bruise percentage in pear. Also, in any storage period, the bruise percentage was intensified with an increase in the loading force. In Figure 7, the maximum and minimum percentages of bruises are 45.138 and 0.094, respectively, belonging to storage periods of 15 and 5 days and loading pressures of 130 and 70 N. According to these results, when a fruit is pressed by another fruit, the cells surrounding the pressured area undergo shear or flexural stresses, thereby reducing the tissue hardness of the fruits and increasing the bruise. During a compression test of a premature or mature fruit, the cell demonstrated the response of this reaction mechanically within itself, leading to a deformation at the compression site after a while (Li, Miao, & Andrews, 2017). Razavi et al. (2018) reported similar results related to the effect of loading and mechanical properties on Pear (Razavi et al., 2018).

### 3.2 The effect of thin edge pressure on bruise percentage

According to Table 1, the thin edge pressure in the storage periods and the loading force is significant at 1% level. Moreover, Figure 8 indicates the significance of its interaction. Considering the results of the constant loading force, an increase in the storage period leads to a corresponding rise in the bruise percentage in pears. Similarly, under a constant storage period, the increase in the loading force augmented the percentage of the bruise. The highest and lowest bruise percentages were 19.880% and 0.007%, respectively, belonging to the 15‐ and 5‐days storage periods, and 25 and 5 N loading pressures.

### 3.3 The effect of impact on bruise levels

Based on Table 1, the impact mode is significant in terms of storage and loading force at 1% level. Figure 9 illustrates the significance of its interaction effect. At a constant loading force, as the storage period increased, the bruise percentage is increased. Similarly, in a constant storage period, bruise percentage increased with the rise in the loading force. The highest and lowest percentages of bruises were 47.36 and 0.21, respectively, occurring in 15 and 5 days into the storage and under 400 and 300 g mass.

The current results revealed that the mass of 400 g created the highest pressure in the present study. Needless to say, the bruises were higher under these pears compared with the other two masses. The energy absorbed by the pear influenced the quality of the fruit significantly during the process of storage and processing, and caused the bruises and mechanical damages on the tissues of the fruits.

The damages to the cells and fruit tissues have a relationship in cytoplasmic oxidizing enzymes, polyphenol oxidase (PPO), and phenolic content. In the presence of oxygen, the enzymatic oxidation of the damaged cells converts phenolic materials into the quinones that polymerize and form dark/brown pigments in the damaged fruits. The brown pigments on the surface of the affected area are the external signs of bruises or compressions; furthermore, the presence of bruises on the newly‐harvested products affects certain physiological processes remarkably, which is acknowledged as the moisture breathing through the injured skin. In particular, the changes in metabolic processes such as ethylene production, relative electrical conductivity, respiration, and transpiration, more than often, result in a mass loss, aging, corruption, and loss of nutritional value (Hussein, Fawole, & Opara, 2018). These results are in accordance with that of Komarnicki, Stopa, Szyjewicz, and Młotek (2016) on the bruise resistance of pear against impact. They reported that bruises were not visible in small loads; however, they became more and more visible with an increase in the load (Komarnicki et al., 2016). Also, Ahmadi, Ghassemzadeh, Sadeghi, Moghaddam, and Neshat (2010) and Zarifneshat et al. (2010)reported similar results related to the effect of loading and mechanical properties on peach and Golden Delicious apple, respectively (Ahmadi et al., 2010; Zarifneshat et al., 2010).

## 4 CONCLUSIONS

- When a pear is affected by wide edge (pear‐to‐pear) pressure in the shipping box, or in the store or fruit shop, it is best to be utilized within 6–9 days. Wide edge pressures of 70, 100, and 130 N, which are increased in the storage period from 5 to 10 days, lead to bruising rates of 78.73, 2.34, and 2.95 for quasi‐static loading. Regarding the storage periods of 10–15 days, increasing the storage days at 70, 100, and 130 N augments the bruise percentage by 1.30, 0.99, and 1.14 times, respectively. In the storage periods of 5, 10, and 15 days and the loading pressures of 70–100 N, the bruise rates were 55.63, 1.65, and 1.25, respectively, and by increasing the level of a bruise, the bruise rates were 2.56, 3.23, and 3.71, respectively.
- If a pear is affected by thin edge pressure, exerted by the corners of the fruit boxes, it is better to be used within 10 days. At thin edge pressures of 15, 20, and 25 N, with the increase in the storage life from 5 to 10 days, the bruise rates for quasi‐static loadings were 22.71, 1.65, and 2.70 times, respectively. Also, from 10 to 15 days, an increase in the storage days augmented the bruise rates by 2.36, 2.17, and 1.10 times at 15, 20, and 25 N, respectively. In addition, during storage periods of 5, 10, and 15 days, the bruise rates were 57.92, 1.65, and 1.25 at 15 to 20 N, and 1.64, 2.67, and 1.35 at 100 to 130 N, respectively.
- If a pear is affected by an impact from falling down a tree or human treatment, it is best to be used in <15 days. With the increase in the storage period from 5 to 10 days, the bruise percentages at 300, 350, and 400 g loading pressures were 8.79, 1.08, and 2.95, respectively. From 10 to 15 days, increasing the storage days augmented the bruise rates by 2.32, 2.37, and 1.19 times at 300, 350, and 400 g mass, respectively. In the storage periods of 5, 10, and 15 days, for 300–350 g, the bruises were 22.66%, 2.78%, and 2.84%, and for 350–400 g, the bruise rates were 2.81, 7.68, and 387 times, respectively.
- According to the results, the quasi‐static loading has a greater effect on the bruise levels in pears and the highest effect of loading belongs to the wide edge pressure. Also, the best storage period is 5 days during which the pears are least affected regarding their quality. From 10 days onward, no significant change is observed in the bruises with the passage of time.

## CONFLICT OF INTEREST

The authors declare that they do not have any conflict of interest.

## ETHICAL STATEMENT

This work does not involve any human or animal studies.
